# Wood and bark structure in *Buddleja*: anatomical background of stem morphology

**DOI:** 10.1093/aobpla/plad003

**Published:** 2023-01-24

**Authors:** K E Frankiewicz, J H Chau, J Baczyński, A Wdowiak, A Oskolski

**Affiliations:** Institute of Evolutionary Biology, Faculty of Biology, Biological and Chemical Research Centre, University of Warsaw, Żwirki i Wigury 101, 02-089 Warsaw, Poland; Centre for Ecological Genomics and Wildlife Conservation, Department of Zoology, University of Johannesburg, PO Box 524, Auckland Park 2006, Johannesburg, South Africa; Institute of Evolutionary Biology, Faculty of Biology, Biological and Chemical Research Centre, University of Warsaw, Żwirki i Wigury 101, 02-089 Warsaw, Poland; Department of Plant Bioenergetics, Institute of Experimental Plant Biology and Biotechnology, Faculty of Biology, University of Warsaw, Miecznikowa 1, 02-089 Warsaw, Poland; Department of Botany and Plant Biotechnology, University of Johannesburg, PO Box 524, Auckland Park 2006, Johannesburg, South Africa

**Keywords:** Bark anatomy, macroscopic bark structure, wood anatomy, sclerification, Scrophulariaceae taxonomy

## Abstract

Bark (all tissues outside of the vascular cambium) has been extensively studied in recent years, especially its anatomy and physiology. Macromorphological bark characters can be important taxonomically for many plant groups, including the genus *Buddleja* (Scrophulariaceae). However, the relationship between macroscopic bark appearance and its microscopic structure remains obscure, hampering the use and interpretation of bark traits in plant taxonomy and phylogenetics as well as in other fields of botany. We studied micro- and macrostructure of bark in the species of *Buddleja* representing wide taxonomic and geographic diversity to identify general relationships between bark anatomy and morphology. We also examined *Buddleja* xylem and discussed the importance of anatomical traits for understanding the relationships between clades in this genus. The smooth bark surface in sect. *Gomphostigma* and the outgroup (*Freylinia* spp.) relates to the small number of periderms of superficial origin and limited sclerification. This allows for the retention of visible lenticels. In the rest of *Buddleja*, bark sloughs off and division of labour is present: collapsed phloem undergoes sclerification and acts as a protective layer, while thin-walled phellem forms the separation layers. A similar pattern is found in some groups (e.g. *Lonicera*), but in others (e.g. *Vitis* and the species of *Eucalyptus* with stringy bark), the pattern is inversed. Wood and bark anatomy supports a sister relationship between the southern African section *Gomphostigma* and the rest of *Buddleja* but is taxonomically uninformative among remaining clades. Limited development of periderms and sclerification allows for the retention of a smooth bark surface and conspicuous lenticels. Sloughing off of bark requires division of labour into a lignified protective layer and a thin-walled separation layer. These two functions are never served by a single tissue but are rather divided between phloem and periderm. How more subtle features (e.g. size and shape of fissures) are determined requires further study. Simultaneously, bark anatomy could be a useful source of data to complement molecular phylogenetic studies in a total evidence approach for systematics.

## Teaser

We explored the relationship between bark surface appearance and its microscopic structure using butterfly bushes (Buddleja, Scrophulariaceae) as models. Smooth stems with visible lenticles develop when periderms are few, originate superficially, and cells undergo a limited sclerification. Barks that slough off always show division of labour at the anatomical level: some cells become sclerified and protective, others remain thin-walled and act as separation layers. These functions are never served by one tissue. We tentatively linked the anatomy and morphology of bark, but much work is still required to fully understand how other traits (like size of fissures) develop in this plant-environment frontline.

## Introduction

Bark anatomy and physiology have recently received much attention (Angyalossy *et al.* 2016; Rosell 2016; Morris and Jansen 2017; Serra *et al.* 2022), but how anatomical traits translate to the staggering diversity of bark morphologies observed among angiosperms remains unexplored. This may be partly because unlike for anatomy, a terminology proposed for description of the bark morphology has not been commonly accepted (Trockenbrodt 1990; Junikka 1994). Among the few articles on the interdependence between the bark macroscopic appearance and microscopic structure (Chattaway 1953, 1955; Kotina *et al.* 2012, 2017; 2018; Kopanina *et al.* 2022), Whitmore’s studies of Dipterocarpaceae are probably the most important (1962a, 1962b; 1962c). He recognized seven bark types based on the features of the bark surface, sloughing modes, patterns of periderm formation, presence of expansion tissue, and structural traits. We used this classification as a framework and *Buddleja* (Scrophulariaceae) as a model to study the relationship between morphological and anatomical structural levels.

**Figure 1.**
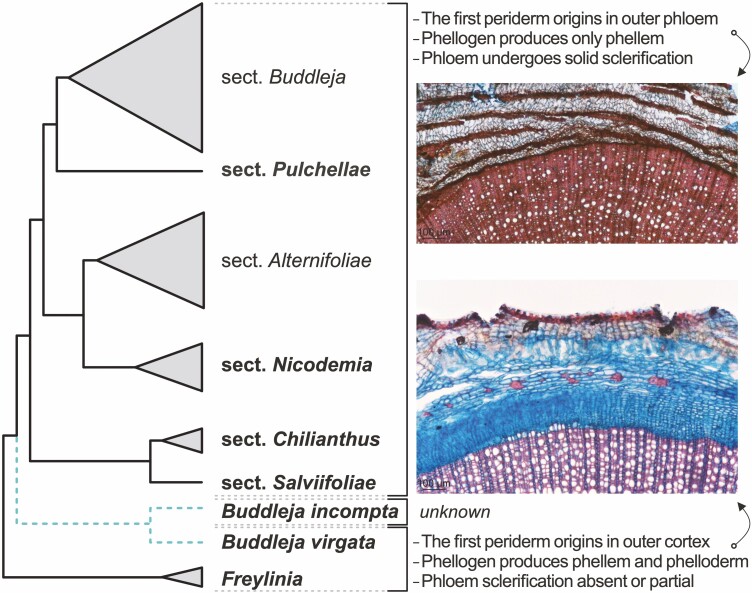
Infrageneric relationships in *Buddleja* and a summary of its bark anatomy. Phylogenetic tree inferred by Chau *et al.* (2017) that was supported by subsequent bark anatomical analyses by Frankiewicz *et al.* (2020), with sect. *Gomphostigma* marked in blue dashed line (representing uncertain position), and African taxa in bold. Only formally recognized clades are named, and clade triangle size approximates species richness, while monospecific clades are solitary lines. The most important anatomical differences between the two types of barks found in *Buddleja* and closely related *Freylinia* reported by Frankiewicz *et al.* (2020) are given at far right. The upper photo is from *B. glomerata* (sect. *Chilianthus*) and represents the more prevalent bark anatomy type. The lower photo is from *B. virgata* (sect. *Gomphostigma*) and depicts the bark anatomy type found only in this species and *Freylinia* in the previous analysis of Frankiewicz *et al.* (2020). The key differences in bark anatomy are listed. The bark anatomy of *B*. *incompta*, the second species in sect. *Gomphostigma*, was previously unknown.

The genus *Buddleja* consists of 108 woody species (Chau *et al.* 2017). Most of the diversity is found in the New World section *Buddleja* (66 species) and Asian sect. *Alternifoliae* (24 species); eight species are Malagasy (together with *B. polystachya* from East Africa and the Arabian Peninsula, they form sect. *Nicodemia*), and the rest is from southern Africa (Fig. 1). Southern African species form a grade of successive sister groups to the rest of *Buddleja*: (1) monospecific sect. *Salviifoliae* (*B. salviifolia*), (2) sect. *Gomphostigma* (with two species: *B. virgata* and *B. incompta*), (3) sect. *Chilianthus* (with four species), (4) monospecific sect. *Pulchellae* (*B. pulchella*; some analyses resolved *B. pulchella* as the sister to the sect. *Buddleja*) and (5) *Buddleja glomerata* of uncertain position (most likely in sect. *Chilianthus*). The sister position to the rest of *Buddleja* is not fully resolved and occupied either by sect. *Salviifoliae* or sect. *Gomphostigma* (Chau *et al.* 2017).

A recent paper (Frankiewicz *et al.* 2020) reported that wood anatomy in *Buddleja* is of little taxonomic use as it is affected by species’ maximal height and does not differ substantially among infrageneric sections. The same paper contained the first study of bark structure in *Buddleja*, including most of the southern African, some Asian and New World representatives, and two outgroups (*Freylinia* spp. from tribe Teedieae sister to Buddlejeae). The examined barks vary considerably (Fig. 1). In *Freylinia* and *Buddleja virgata*—the only representative of sect. *Gomphostigma* available in that study—the first-formed phellogen originates in the outer cortex and produces phellem and phelloderm; secondary phloem may be non-sclerified (*B. virgata*) or sclerification is only partial (*Freylinia*). In the remaining species of *Buddleja*, the first phellogen originates in the outer phloem and subsequent periderms cut off portions of phloem that undergoes solid sclerification; no phelloderm was observed. The similarity of bark structure between *Buddleja virgata* and *Freylinia* supports a sister position of sect. *Gomphostigma* with respect to the rest of *Buddleja*. The lack of phelloderm in all *Buddleja* species (except *B. virgata*) is particularly noteworthy as it implies a shift of cork cambium activity from bifacial to unifacial and would make a rare finding if confirmed (Evert 2006).

The present article aims to reignite interest in the anatomical–morphological relationship in bark which has received little attention since the 1960s. We undertake one of the very first attempts to describe bark macroscopic appearance in a systematic way, covering most of the taxonomic diversity and geographic range of *Buddleja*. Next, we examine wood and bark anatomy of 14 previously unstudied species and compare them with those previously reported in the literature. Finally, we discuss how anatomical traits interact to result in the staggering diversity of bark morphologies observed in woody plants and how anatomical traits contribute to current understanding of the systematics of *Buddleja*.

## Methods

### Origin of specimens

Sixteen species of *Buddleja* (Buddlejeae, Scrophulariaceae; 19 specimens) were sampled: 14 species (17 specimens) were obtained from plants cultivated in the National Buddleja Collection at Longstock Park Nursery (Stockbridge, Hampshire, United Kingdom), one specimen of *B. incompta* came from a natural population (Sutherland, Northern Cape, South Africa), and a sample of *B. saligna* was obtained from a cultivated tree (Johannesburg, Gauteng, South Africa). Fourteen species were studied for the first time, two were re-examined (Frankiewicz *et al.* 2020).

The taxonomic coverage of new samples included: one species from sect. *Gomphostigma* (*B. incompta*), one species from sect. *Chilianthus* (*B. saligna*), three species from the New World sect. *Buddleja*, nine representatives of Asian sect. *Alternifoliae*, and two hybrids. Herbarium vouchers were deposited in JRAU, NBG or WA (Thiers 2013) and their photos were uploaded online (Bianchi and Gonçalves 2021), accession details are given in Appendix A (see ‘Data’ and ‘Supporting Information’).

### Anatomical study

For wood anatomical studies, samples were obtained from the thickest lateral branch or a main stem. For bark examination, three pieces were cut: young twigs without visible periderm, lower parts of branches where periderm was starting to form, and thick branches with well-developed periderm. Material was immediately stored in 70% ethanol.

Standard anatomical procedures were followed (Frankiewicz *et al.* 2020): we cut snippets of wood and bark 20–40 µm thick with sledge or freezing microtome, stained them with safranin and alcian blue, and mounted in Euparal. Histochemical tests for lignin and lipides presence were performed for the bark sections (Johansen 1940). To check the details of periderm structure, two specimens (*B. incompta* and *B. saligna*) were embedded in GMA, sectioned on an ultramicrotome, stained with toluidine blue, and mounted in Entellan (Feder and O’Brian 1968). Length of vessel elements and fibres was measured from macerated material. Wood descriptions and measurements follow the IAWA Committee (1989) and bark descriptions are according to Angyalossy *et al.* (2016). Measurements are available online (see ‘Data’).

### Macroscopic bark descriptions

Aside from anatomical descriptions, we described macroscopic bark features (surface morphology). A universally accepted terminology of bark morphology is missing—we followed the most comprehensive nomenclature system presented by Junnika (1994).

In total, we studied 26 species (32 specimens) from tribe Teedieae (the sister to Buddlejeae, represented by *Freylinia*) and all sections within *Buddleja* except for sect. *Nicodemia* and sect. *Pulchellae* (available samples were too young): we included almost all specimens sampled for the present study (*B. fallowiana* KF037 and *B. myriantha* KF030 were excluded due to moderate bark development) and most specimens examined anatomically by Frankiewicz *et al.* (2020). Appendix A gives the full list of specimens. Bark morphologies were studied with the naked eye and with a dissecting microscope under low magnification; the barks were slashed with a pen knife to check for firmness.

## Results

### Macroscopic bark features of *Buddleja*

**Figure 2.**
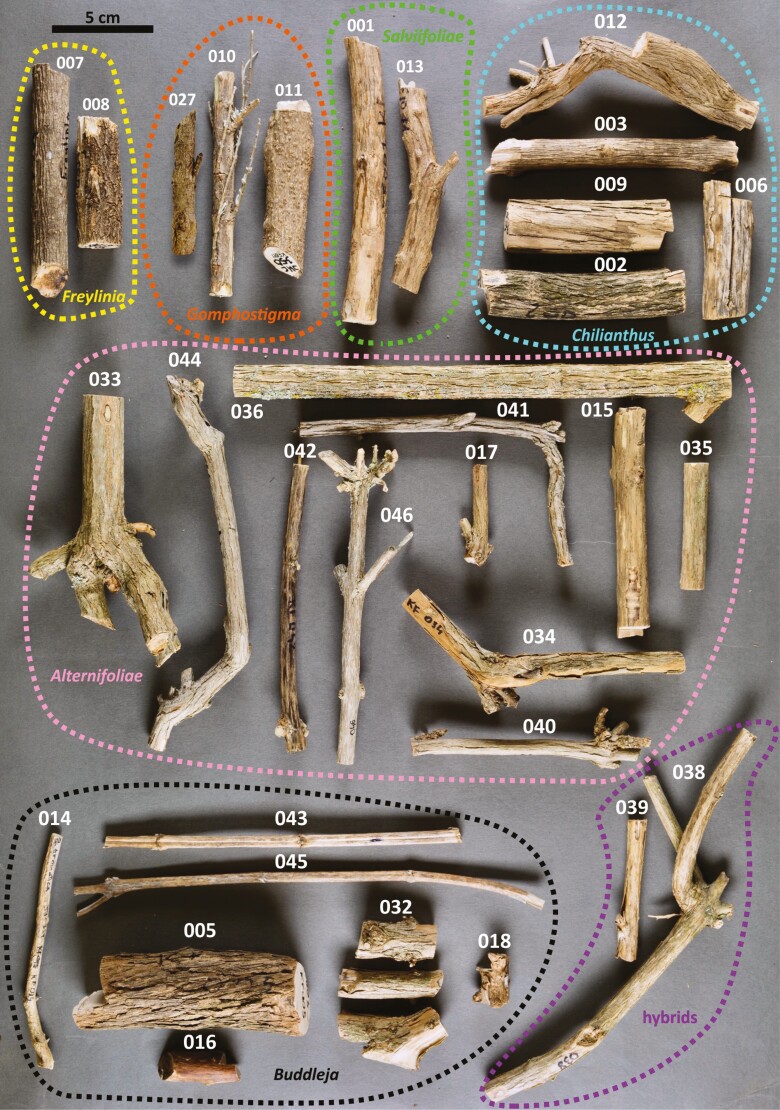
Macroscopic bark appearance of *Freylinia* and *Buddleja*. This figure provides an overview of morphology of all examined samples with collector’s numbers (for accession details see Appendix A). Specimens are arranged taxonomically by clade. Outgroup: (007) *Freylinia lanceolata*, (008) *F. tropica*. Section *Gomphostigma*: (010–011) *B. virgata*, (027) *Buddleja incompta*. Sect. *Salviifoliae*: (001 & 013) *B. salviifolia*. Sect. *Chilianthus*: (002) *B. saligna*, (003 & 012) *B. auriculata*, (006) *B. glomerata*, (009) *B. dysophylla*. Sect. *Alternifoliae*: (015) *B. lindleyana*, (017 & 034 & 040) *B. forrestii*, (033) *B. albiflora*, (035) *B. nivea*, (036) *B. paniculata*, (041) *B. colvilei*, (042) *B. crispa*, (044) *B.* cf. *curviflora,* (046) *B. crispa*. Sect. *Buddleja*: (005) *B. cordata*, (014) *B. aromatica*, (016) *B. coriacea*, (018) *B. longiflora*, (032) *B. globosa*, (043) *B. crotonoides*, (045) *B. tubiflora*. Hybrids: (038) *B.* x *weyeriana*, (039) *B.* x *wardii*. Scale = 5 cm.

**Figure 3.**
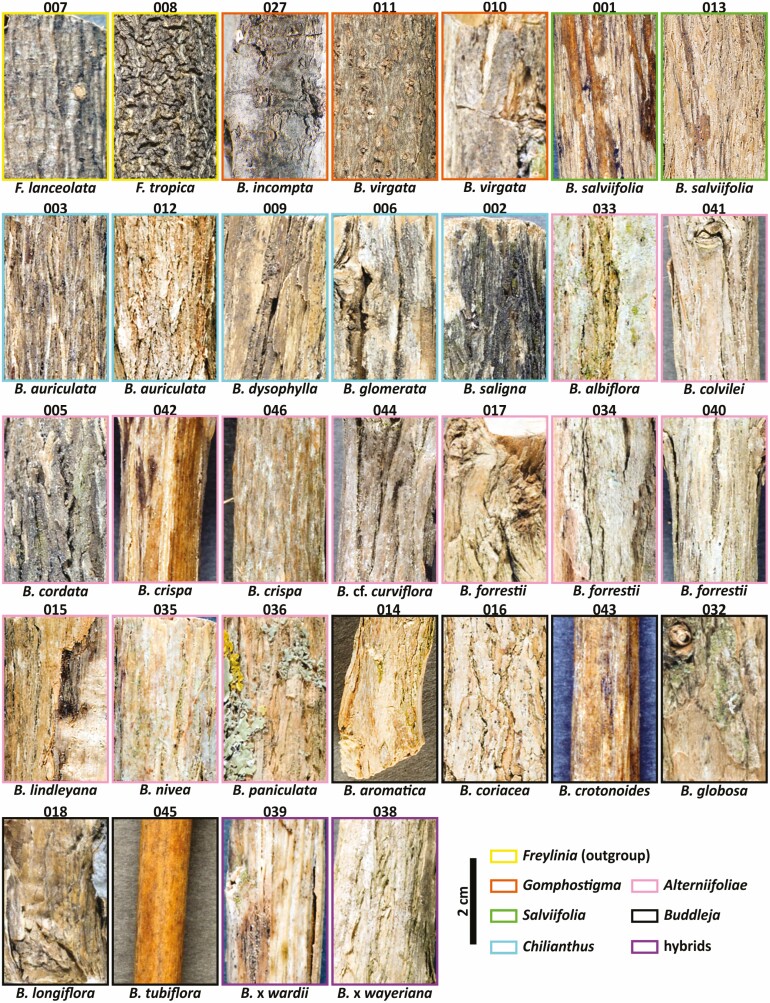
Details of macroscopic bark appearance of *Freylinia* and *Buddleja* species. The specimens are arranged taxonomically by clade as in Figs. 1–2. The collector’s numbers and colour coding are the same as in Figure 2. For accession details see Appendix A. Scale = 2 cm.

**Figure 4.**
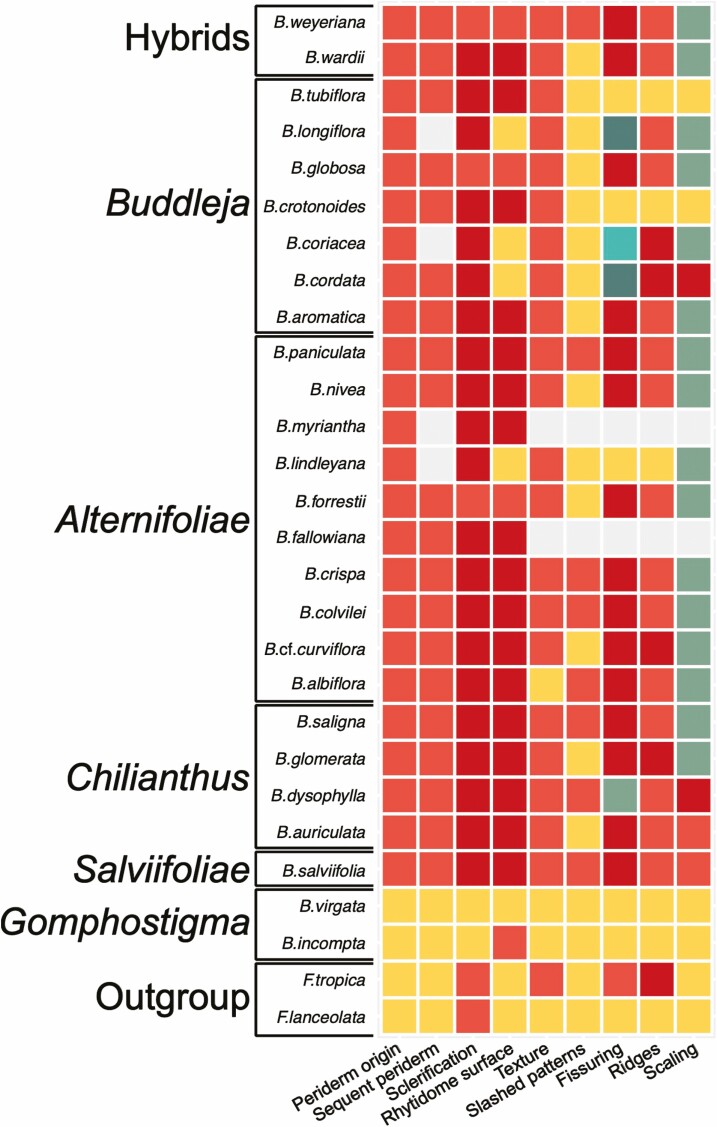
Summary of selected bark anatomical and morphological traits of *Freylinia* and *Buddleja*. This figure shows overall similarity in bark structure among studied groups. The full table on which this figure was based is available as Appendix B. Clade names are given on the far left, and the same taxonomic order as in Figure 1 was retained. The studied traits and their states are: periderm origin: subepidermal (yellow) or in outer phloem (light red); arrangement and origin of sequent periderm: reticulate (yellow) or concentric to reticulate (light red); sclerification pattern of nonconducting phloem: none (yellow), partial with sclereids in small clusters (light red) or nearly solid with sclereids in bands (dark red); structures covering rhytidome surface: rhytidome absent (yellow), collapsed secondary phloem (light red), sclerified secondary phloem (dark red); bark texture: homogenous (yellow) or loose (light red); visual slashed patterns: missing (yellow) or ripple marks (light red); fissuring: absent (yellow), compound (light red), boat-shaped (dark red), oblique (light green), irregular (dark green), wavy (bluish); ridges: absent (yellow), flattened (light red), reticulate (dark red); scaling: absent (yellow), loose-hanged (light red), scaly flakes (dark red), irregular (light green). Missing values are in grey.

Figure 2 gives an overview of bark morphology of all examined species, and Fig. 3 shows close-ups—in both cases specimens are arranged taxonomically, the same colour-coding was applied, and accession numbers in the text and figures allow for cross-referencing. The major bark anatomical and morphological traits are summarized taxonomically in Fig. 4 (the data matrix used to generate this figure is available in Appendix B), and detailed descriptions follow below.

Bark texture is loose (crumbling when slashed) in most species. In *B. albiflora* (KF033), *B. incompta* (KF027), a thick stem of *B. virgata* (KF011) and *Freylinia lanceolata* (KF007) it is homogenous. In thin stems of *B. forrestii* (KF040) and *B. tubiflora* (KF045, very limited secondary growth), bark texture is also homogenous—likely because multiple periderms have not yet developed. In a thinner stem of *B. virgata* (KF010), the stem bark is rather homogenous and crumbles only slightly when slashing.

Bark patterns (when slashed tangentially) are missing, except in *B. albiflora* (KF033), *B. dysophylla* (KF009), *B. crispa* (KF042), *B. colvilei* (KF041) and *B. saligna* (KF002), which have distinct ripple marks, and *B. paniculata* (KF036), *B. salviifolia* (KF013) and *B.* x *weyeriana* (KF038), where ripples are indistinct.

Fissuring is present in most species. In *B. crotonoides* (KF043) and *B. tubiflora* (KF045), fissures are macroscopically absent but observed under binoculars (possibly due to the specimens’ limited secondary growth). In four species, they are absent or very rare: in *B. lindleyana* (KF015), the stem is smooth; in *F. lanceolata* (KF007), *B. incompta* (KF027) and a thick stem of *B. virgata* (KF011), the surface is only ruffled (in the two latter, it is sometimes broken transversely, not parallel to stem length). A thinner stem of *B. virgata* (KF010) resembles *B. incompta* with slight fissuring/ruffling (the thicker stem of *B. virgata* [KF011] has a much smoother appearance).

Fissuring is of six types: (1) boat shaped (in *B. albiflora* [KF033], *B. aromatica* [KF014], *B. auriculata* [KF003, KF012], *B. forrestii* [KF034], *B. globosa* [KF032], *B. saligna* [KF002], *B. salviifolia* [KF001, KF013] and possibly also in *B. crispa* [KF046]); (2) parallel to boat shaped (in *B. crispa* [KF042], *B.* cf. *curviflora* [KF044], *B. colvilei* [KF041], *B. forrestii* [KF040], *B. glomerata* [KF006], *B. paniculata* [KF036], *B. nivea* [KF035], *B.* x *weyeriana* [KF038], *B.* x *wardii* [KF039]); (3) parallel to oblique (in *B. dysophylla* [KF009], possibly also in *B. forrestii* [KF017]); (4) wavy (*B. coriacea* [KF016]); (5) irregular (*B. cordata* [KF005], *B. longiflora* [KF018]); or (6) compound (*F. tropica* [KF008]).

Ridges are absent in species without fissuring: *F. lanceolata* (KF007), *B. incompta* (KF027), *B. virgata* (KF010, KF011), *B. lindleyana* (KF015), *B. crotonoides* (KF043), *B. tubiflora* (KF045); flattened in all remaining species except *B. cordata* (KF005), *B.* cf. *curviflora* (KF044), *B. coriacea* (KF016), *B. glomerata* (KF006, and *F. tropica* (KF008) where they are reticulate.

Scaling is absent in *Freylinia* (in *F. tropica*, some tendency towards chunky scales is visible), the thick stem of *B. virgata* (KF011), *B. incompta* (KF027), *B. crotonoides* (KF043) and *B. tubiflora* (KF045). In the remaining species, flakes are scaly (*B. cordata* [KF005], *B. dysophylla* [KF009], *B. auriculata* [KF012]) or irregular. In *B. auriculata* (KF012) and *B. salviifolia* (KF013), it is loose-hanged at the same time or even stringy.

External markings are well seen only in *F. lanceolata* (KF007) and *B. virgata* (KF011), where the bark is lenticellate with lenticels round, scarce, solitary and medium in size. Additionally, the bark surface can be rough (*B. cordata* [KF005]), smooth (*B. crotonoides* [KF043], *B. tubiflora* [KF045]), or rugose (in the remaining species; in *F. lanceolata* [KF007], the bark is distinctly wrinkled, but the surface is not broken).

### Bark anatomy of *Buddleja incompta*

**Figure 5.**
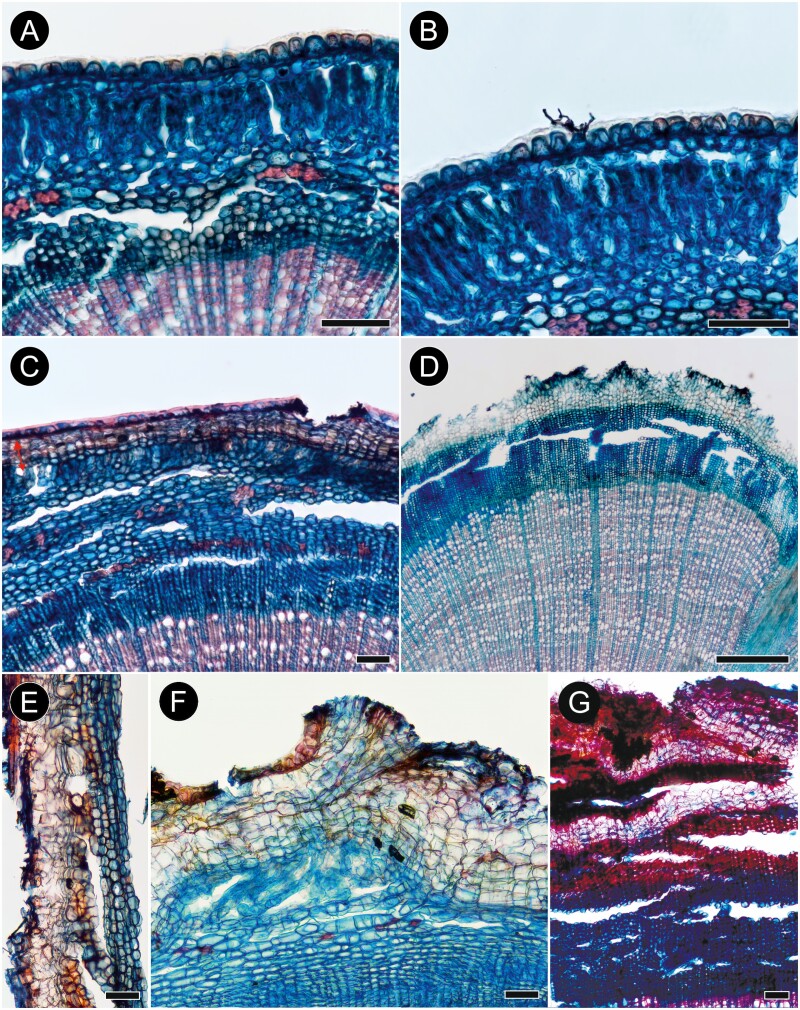
Bark anatomy of sect. *Gomphostigma*: *Buddleja incompta* (A–E; G) and *B. virgata* (F) in transverse (A–D, F) and radial (E) sections. (A) Young stem prior to initiation of periderm. Dome-like cells of epidermis are followed by a layer of isodiametric cells and a few layers of radially elongated cells with wide spaces between them. Pericycle is parenchymatous with discrete strands of fibres, and secondary phloem is homogenous. (B) Details of dome- and bottle-like epidermis cells covered with prominent cuticle, and possible remnants of a trichome in the centre. (C) Early stages of initiation of the first periderm (marked with red arrow on the left). The first phellogen forms just beneath the epidermis—which is still present and clearly visible—and produces phellem and phelloderm. Some sclerification occurs within the primary cortex, but not in the secondary phloem. (D) A subsequent periderm formed close to the secondary phloem. Despite the advanced stage, no phloem sclerification is present. (E) Formation of the first periderm in the outer cortex; the stem pith in this photo is on the right, the inset shows periderm detached from the stem with two abnormal periderms formed obliquely. (F) Formation of the first periderm in *B. virgata* studied in (Frankiewicz *et al.* 2020): the first phellogen forms in the outer cortex and no sclerification is present except in rare, solitary cells. (G) Rhytidome with three periderms in *B. incompta*. Scale bars: D = 500 µm, A–C & E–G = 100 µm.

The epidermis is composed of a single layer of bottle- to dome-like cells, (16–)25(–34) µm in tangential size with thin walls and prominent cuticle (Fig. 5A). Trichomes were not observed.

The cortex is composed of ≤10 cell layers: the outermost layer, directly adjoining the epidermis, consists of isodiametric cells; it is followed by a few layers of radially elongated, palisade cells with large, intercellular spaces, and the innermost layers are made up of isodiametric, rounded cells (17–)27(–37) µm in tangential diameter (Fig. 5B). The pericyclic fibres are very thick-walled, mostly in small groups of 4–8 (rarely ≤17), sometimes solitary (Fig. 5C). No crystals were observed in cortical cells. Dilatation of the cortex is affected by tangential stretching of cortical cells and anticlinal divisions of parenchyma cells leading to tangential strands of 2 cells (Fig. 5B, C).

**Figure 6.**
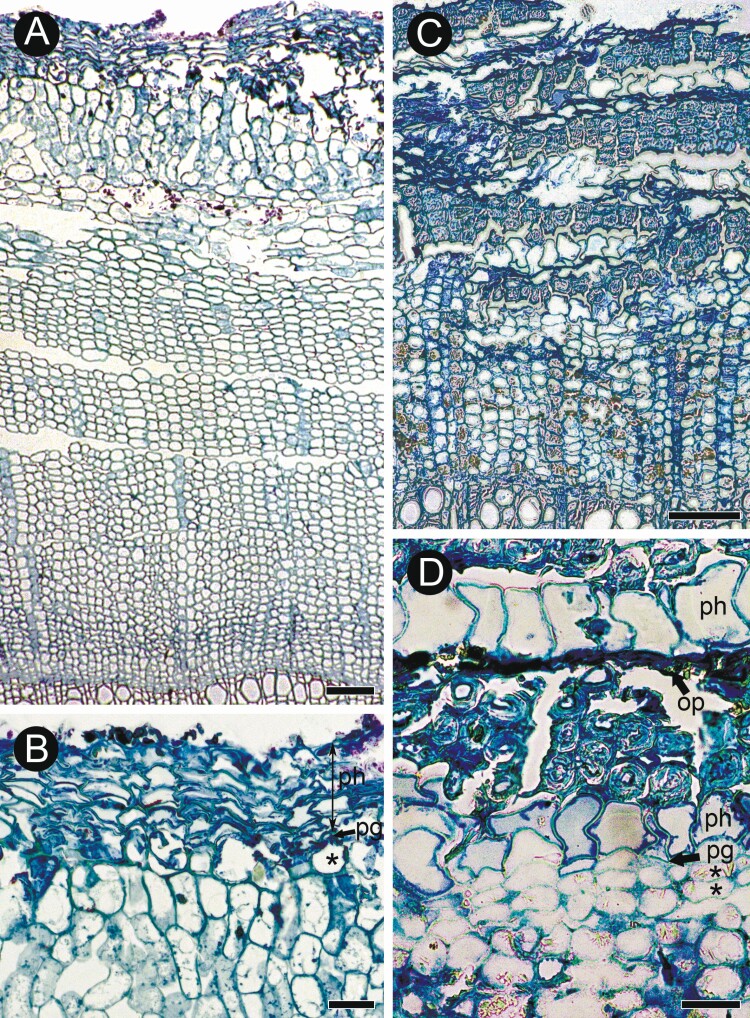
Details of phelloderm formation in *Buddleja incompta* (sect. *Gomphostigma*; A, B) and *B. saligna* (sect. *Chilianthus*; C, D) on transverse sections. (A) Bark of *B. incompta* with broad homogenous phloem and the periderm formed outside the cortex. (B) Details of periderm in *B. incompta*: phellem (ph), phellogen (pg), phelloderm (asterisk). (C) Bark of *B. saligna* with homogeneous conducting phloem, rhytidome with five periderms and portions of non-conducting phloem affected by nearly solid sclerification. (D) Details of recently formed periderm, older periderm and non-conducting phloem in *B. saligna*: phellem (ph) in both periderms consist of a single layer of thin-walled (phelloid) cells; phellogen (pg) and two layers of phelloderm (asterisks) found only in the recently formed periderm, and obliterated in the older one; the band of non-conducting phloem is sclerified in its inner zone and obliterated (op) in the outer zone. Scale bars: A, C = 100 µm, B = 50 µm, D = 20 µm.

The first-formed periderm is initiated in the subepidermal layer of the outer cortex. The subsequent periderms are initiated in secondary phloem, and their arrangement is reticulate. Phellem is non-stratified and consists of ≤10 layers of isodiametric to tangentially elongated phelloid cells (Fig. 5D–G) with weakly lignified non-suberized walls. Phelloderm is uni- or biseriate (Fig. 6A, B). The rhytidome is present, its outer surface is covered by collapsed secondary phloem (Fig. 5G).

Conducting secondary phloem is very homogenous and consists of sieve tubes with companion cells (typically one per sieve tube member in cross section), axial parenchyma, and rays (Fig. 5D–F). Sieve tube members are solitary or in small groups, (8–)13(–18) µm wide in tangential diameter and (86–)111(–165) µm long. Sieve plates are compound with 4–10 sieve areas on strongly inclined walls. Axial parenchyma forms the ground tissue and consists of fusiform cells and strands of ≤ 5 cells. Transition from conducting to nonconducting phloem is gradual, marked by tangential stretching of phloem elements. The nonconducting phloem consists of weakly obliterated and heavily collapsed elements; sclerified elements were not found.

Secondary phloem rays are uni- and biseriate. They are composed of square, procumbent and some upright cells forming multiple rows (≤10) flanked by upright cells, or all types of cells are intermixed throughout the ray body. No crystals, tannin deposits or secretory structure were observed.

Dilatation of secondary phloem is affected by tangential stretching of sieve tube members, axial parenchyma cells and ray cells, but no anticlinal divisions were found; possibly the broad intercellular spaces in the outer cortex are also the result of dilatation. No sclerification of any secondary phloem elements was observed.

### *Bark anatomy of other* Buddleja *species*

Details of young stem anatomy before the development of periderm were observed in *B. albiflora*, *B. crispa*, *B. crotonoides*, *B. fallowiana*, *B. forrestii*, *B. globosa*, *B. myriantha*, *B. nivea*, *B. paniculata*, *B.* x *wardii* and *B.* x *weyeriana*. The bark structure in stems with fully developed periderm was observed in all the previous species, as well as in *B.* cf. *curviflora*, *B. colvilei* and *B. tubiflora*.

**Figure 7.**
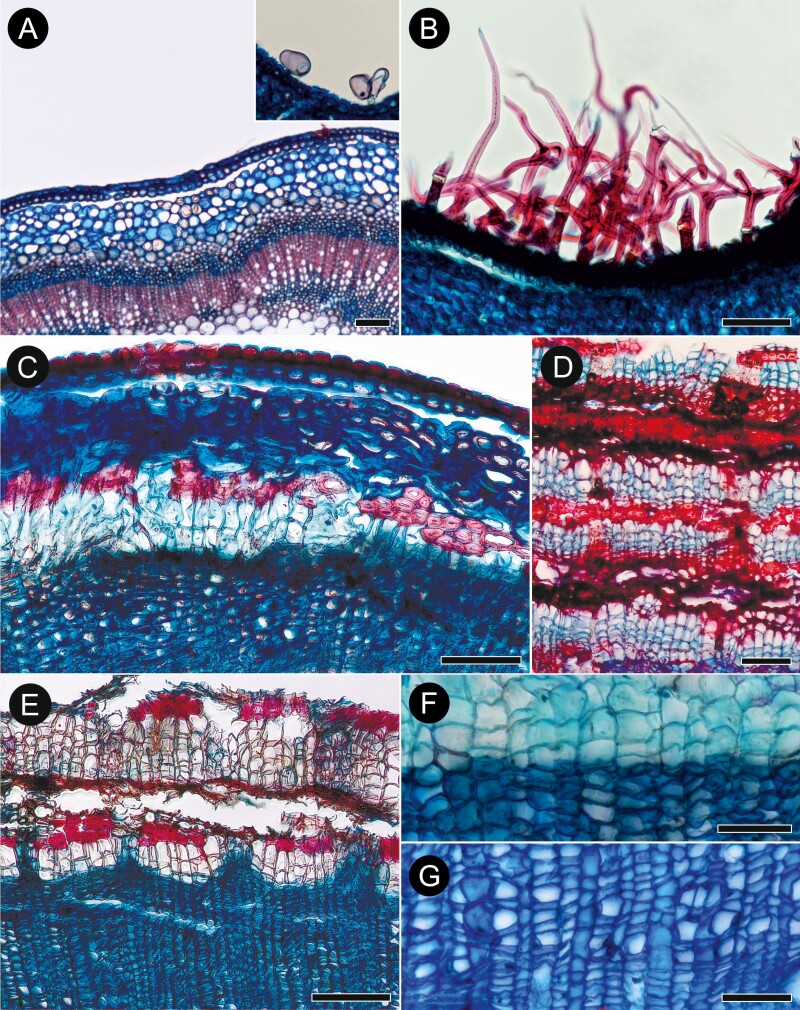
Bark anatomy of selected *Buddleja* species in transverse sections. (A) Glabrous young stem of *B. albiflora* (sect. *Alternifoliae*) with very inconspicuous cuticle; inset shows glandular hairs of *B.* x *weyeriana*; (B) Dense indumentum of branched trichomes in *B. paniculata* (sect. *Alternifoliae*); (C) A stem of *B. albiflora* with first-formed periderm initiated inside of pericycle; (D) At least four periderms developed in *B. albiflora*, each consisting of phellogen and phellem composed of thin-walled cells; between periderms are portions of cut-off phloem with collapsed phloem elements and continuous bands of sclereids; (E) Portion of secondary phloem and rhytidome in *B. globosa*; sclereids in the portions of cut-off phloem in small clusters and interrupted bands; (F) Border between secondary phloem (lower half) and phellem (upper half) in *B. albiflora*; phellogen cells can be recognised (unlike phelloderm); (G) Details of secondary phloem in *B. globosa* (sect. *Buddleja*) showing numerous solitary sieve tube members and some that are grouped in tangential bands, each with single companion cell. Scale bars: A–E = 100 µm, F–G = 50 µm.

The epidermis is uniseriate, composed of isodiametric (*B. albiflora*, *B. crotonoides*, *B. fallowiana*, *B. forrestii*, *B.* x *wardii*), tangentially elongated (*B. crispa*, *B. globosa*, *B. myriantha*, *B. nivea*, *B. paniculata*), or intermediate (*B.* x *weyeriana*) cells (Fig. 7A). The epidermal cells are (14–)15–21(–23) µm in tangential diameter, their walls are thin, and cuticle is not prominent. Trichomes were not observed in *B. albiflora* (Fig. 7A), *B. crotonoides*, and *B. myriantha*; in the remaining species, rare, branched trichomes were present (*B. crispa*, *B. fallowiana*, *B. forrestii*, *B. globosa*, *B. tubiflora*, *B.* x *wardii*) or trichomes were numerous (*B. nivea*, *B. paniculata*; Fig. 7B); few glandular trichomes were found in *B. crotonoides*, *B. forrestii*, *B. globosa*, *B. nivea*, *B. paniculata*, *B.* x *wardii* and *B.* x *weyeriana* (Fig. 7A inset).

The cortex is composed of parenchymatous cells (6–8 layers in *B. albiflora*, ≤10 layers in *B. crispa*, *B. crotonoides*, *B. fallowiana*, *B.* x *wardii*, and *B.* x *weyeriana*; ≤15 in *B. forrestii*, c. 10 in *B. globosa*, c. 6 in *B. myriantha*, ≤12 in *B. nivea*, ≤15 in *B. paniculata*; Fig. 7A). The cells are tangentially stretched or isodiametric and rounded, and (15–)17–30(–35) µm in tangential diameter. No pericyclic fibres were found in *B. paniculata* and *B.* x *wardii*; in the remaining species, moderately thick-walled pericyclic fibres are disposed as discrete groups of ≤12 cells or continuous, bi- to pentaseriate ring (Fig. 7C). In *B. crispa* [KF042], *B. globosa* [KF032], *B. nivea* and *B.* x *weyeriana*, pericyclic cells had thickened cell walls, but lignification was not detected.

**Figure 8.**
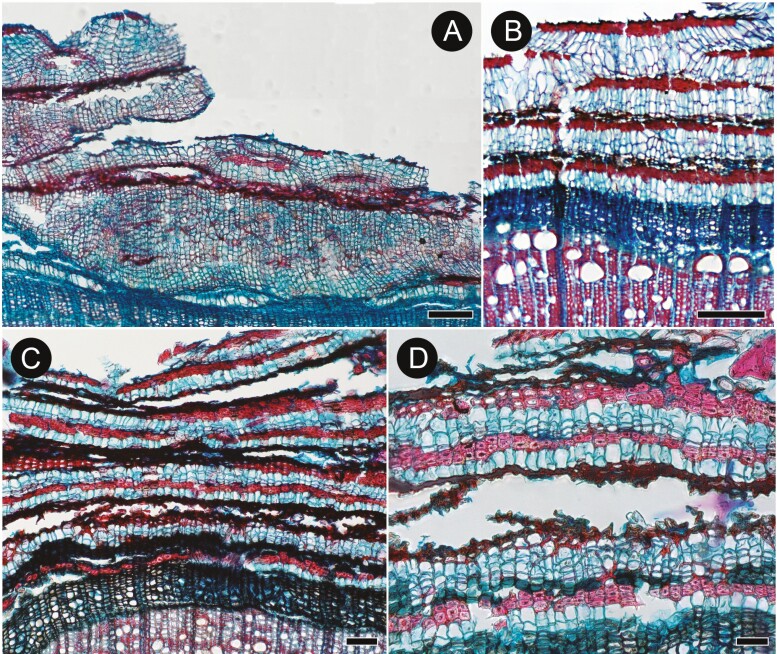
Rhytidome anatomy in selected *Buddleja* species in transverse sections. (A) Exceptionally broad bands and clusters of thin-walled phellem and few sclereids in collapsed secondary phloem, outer surface covered with collapsed phloem in *B.* x *weyeriana*; (B) Phellem bands with radially elongated cells, continuous bands of phloem sclereids (also on the rhytidome surface) in *B. crispa* (sect. *Alternifoliae*); (C) Bands of ruptured collapsed phloem without sclereids alternating with the bands of sclerified phloem in *B. nivea* (sect. *Alternifoliae*); (D) Details of rhytidome in *B. nivea*: some radial files of phloem sclereids are not collinear to the underlying files of phellem cells. Scale bars: A = 200 µm, B & C = 100 µm, D = 50 µm.

The first-formed periderm is initiated in the outermost layer of secondary phloem (Fig. 7C) with concentric to reticulate arrangement of subsequent periderms in deeper layers of secondary phloem (Fig. 7D, E). The mature periderm consists of easily identifiable phellem and phellogen (Fig. 7D–F). Uni- to biseriate phelloderm was recognised only in the youngest (innermost) periderm in GMA microsections of *B. saligna*; and it is obliterated in subsequent periderms (Fig. 6C, D). Therefore, phelloderm most likely is produced in all studied species and undergoes rapid obliteration (Fig. 7F). Phellem is composed of layers (4–9 in *B. albiflora* [Fig. 7D]; 2–3 in *B.* cf. *curviflora*; 3–6 in *B. colvilei*; 2–3 in *B. crispa* [Fig. 8B], *B. crotonoides*, and *B. fallowiana*; 3–5 in *B. forrestii* KF034 and 2–3 in *B. forrestii* KF040; 3–7 in *B. globosa* KF031 and 2–5 in *B. globosa* KF032 [Fig. 7E]; 3–4 in *B. myriantha*; 1–3 in *B. nivea* [Fig. 8C–D]; 2–3 in *B. paniculata* 1–3 in *B. tubiflora*; 1–2 in *B.* x *wardii*; ≤25 in *B.* x *weyeriana*) of isodiametric to radially elongated (Fig. 8B), thin-walled (phelloid) cells with weakly lignified non-suberized walls. The rhytidome is present, its outer surface is covered mostly by collapsed secondary phloem in *B. forrestii*, *B. globosa* (Fig. 7E), *B*. x *weyeriana* (Fig. 8A), or mostly by sclerified secondary phloem in other species.

**Figure 9.**
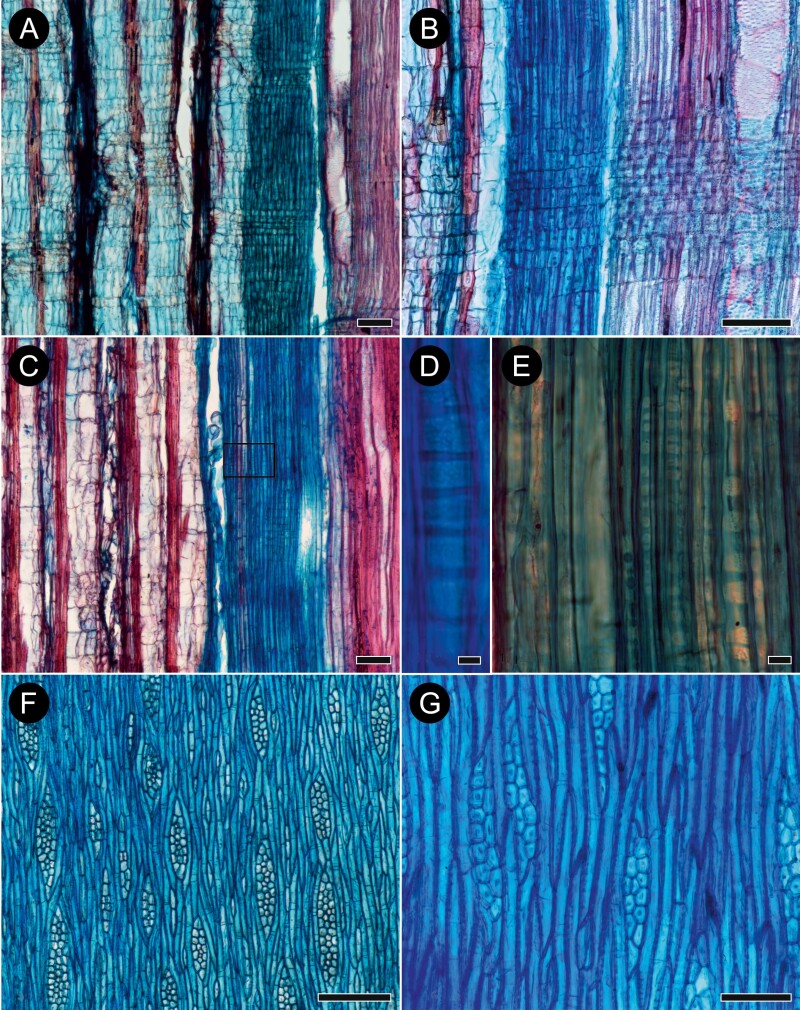
Bark anatomy of selected *Buddleja* species in radial (A–E) and tangential longitudinal (F–G) sections. (A) From right to left: secondary xylem, secondary phloem with two rays well-seen in radial view and five subsequent periderms in *B. albiflora* (sect. *Alternifoliae*); (B) The same order as in (A), showing details of vessel—ray pitting and ray in secondary phloem of *B. nivea* (sect. *Alternifoliae*); (C) *B. paniculata* (sect. *Alternifoliae*) showing the same structures as in (A, B); sieve-plates area where a few sieve plates are seen is marked with a rectangle, and details of compound sieve areas on oblique end walls are in (D) and (E); (F) Numerous, bi- to tetraseriate rays and a few uniseriate rays embedded in axial parenchyma of *B. albiflora* (sect. *Alternifoliae*); (G) Phloem rays with thickened cell walls in *B.* x *weyeriana*. Scale bars: A–C & G = 100 µm, D–E = 10 µm, F = 200 µm.

Conducting secondary phloem is homogenous and consists of sieve tubes with companion cells (typically one per sieve tube member in cross section), axial parenchyma, and rays (Fig. 7G). Sieve tube members are solitary or in small clusters, rarely in tangential bands. Sieve tube members are (12–)13–19(–20) µm wide and (107–)118–174(–186) µm long. Sieve plates are compound with sieve areas located on strongly inclined end walls (5–9 plates in *B. albiflora*, 4–8 in *B.* cf. *curviflora*, 6–9 in *B. colvilei* and *B. crispa*, 3–6 in *B. forrestii*, 6–11 in *B. globosa*, 6–13 in *B. nivea*, 4–12 in *B. paniculata*, 4–8 in *B. tubiflora* and *B.* x *wardii*, 5–10 in *B.* x *weyeriana*; sieve plates were not observed in *B. fallowiana*; Fig. 9D–E). Axial parenchyma forms the ground tissue and consists of fusiform cells and strands of 2–4 in *B. albiflora*, *B.* cf. *curviflora* and *B. colvilei*, 2–5 in *B. crispa* and *B. forrestii*, 2–4 in *B. globosa*, 2–6 in *B. nivea* and *B. tubiflora*, and 4–5 in *B.* x *weyeriana* (Fig. 9F–G); in the remaining species, tangential sections were unavailable. Crystals and other deposits were not found except for droplets of tannin in various cells of *B. colvilei* and ray cells of *B. nivea* (Fig. 9B), and inulin crystals in *B. crispa* (KF046) and *B. forrestii*. Section quality did not allow for examination of the transition zone between conducting and nonconducting phloem in *B. fallowiana* and *B.* x *wardii*. In the remaining species, the transition is sharp, typically marked by periderm initiation. The nonconducting phloem consists of weakly obliterated to heavily collapsed elements, and of the sclerified elements. Zone of phloem sclerification is commonly adjacent to the underlying periderm. *B. forrestii*, *B. globosa* (Fig. 7E) and *B*. x *weyeriana* (Fig. 8A) show tangential bands and lens-like clusters of collapsed phloem elements, occasionally (rarely in *B*. x *weyeriana*) with sclereids in small groups and interrupted 1–2(–3)-seriate bands. Other species (except the juvenile sample of *B. fallowiana*) show more abundant (occasionally nearly solid) sclerification of nonconducting phloem with the sclereids in continuous bands ≤3 cells in width (Fig. 7B–D).

Secondary phloem rays are uni- to tetraseriate, most often bi- to triseriate (Fig. 9F, G). Ray bodies are composed of ≤10 rows of square (with some procumbent and upright) cells flanked by upright cells in one or two rows in *B. albiflora* (Fig. 9A), ≤15 rows in *B.* cf. *curviflora*, ≤28 in *B. crispa*, ≤20 in *B. forrestii*, ≤17 in *B. globosa*, ≤11 in *B. nivea*, ≤15 in *B. paniculata*, ≤10 in multiseriate portions of rays in *B. tubiflora*, and ≤15 in *B.* x *weyeriana* (Fig. 9B, C), or by mixed cell types (in ≤12 rows in *B. colvilei*).

Weak dilatation of secondary phloem affected by tangential cells stretching axial parenchyma and sieve tube elements, usually without anticlinal divisions, was found in *B. albiflora*, *B. myriantha, B. nivea*, *B. paniculata* and *B.* x *weyeriana*; in the remaining species dilatation was not observed. In all cases, conductive cells, axial parenchyma, and rays in the portions of secondary phloem separated by periderms undergo sclerification (1–4 layers in *B. albiflora*, 2–5 in *B.* cf. *curviflora*, 1–5 in *B. colvilei*, 1–3 in *B. crispa*, 2–8 in *B. crotonoides*, 1–5 in *B. forrestii*, 3–7 in *B. globosa*, 2–3 in *B. myriantha* and *B. nivea*, 2–4 in *B. paniculata*, 3–5 in *B. tubiflora*, 2–4 in *B.* x *weyeriana*; Fig. 7D, E, 8A–C).

### Wood anatomy

Wood descriptions are based on samples of *Buddleja albiflora*, *B.* cf. *curviflora*, *B. colvilei*, *B. crispa*, *B. crotonoides*, *B. fallowiana*, *B. forrestii*, *B. globosa*, *B. incompta*, *B. myriantha*, *B. nivea*, *B. paniculata*, *B. tubiflora*, *B.* x *wardii* and *B.* x *weyeriana*. The sample of *B. saligna* was excluded because this species was already described (Frankiewicz *et al.* 2020).

**Figure 10.**
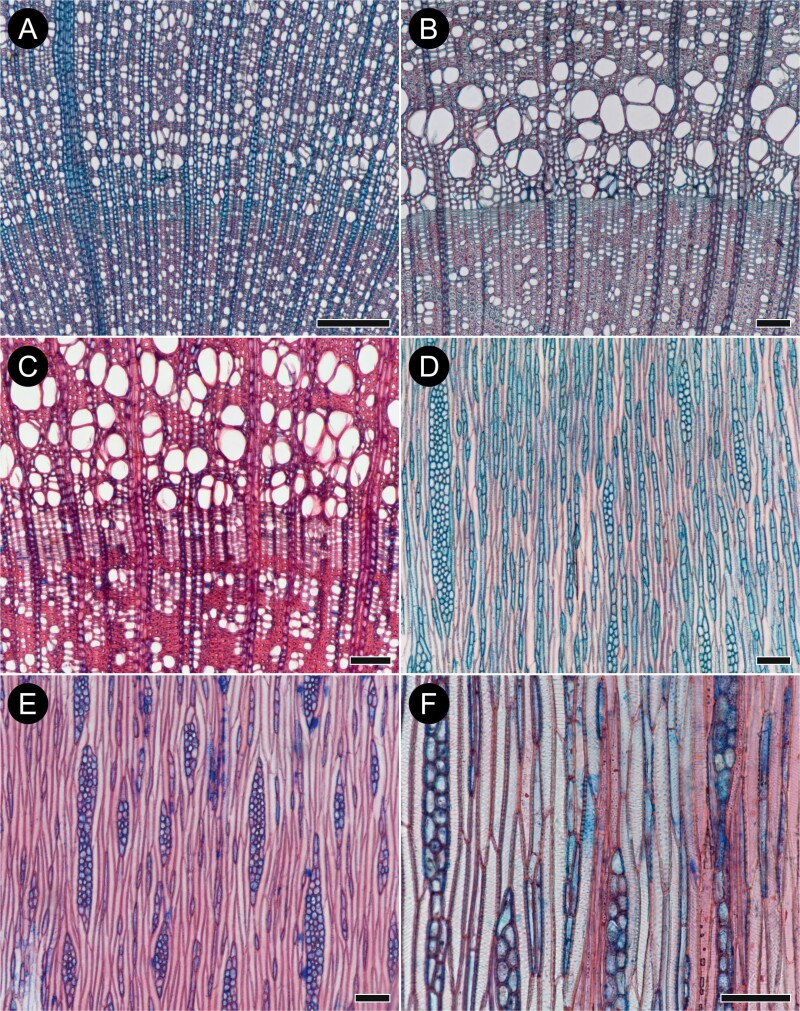
Wood anatomy of selected *Buddleja* species in transverse (A–C) and tangential longitudinal (D–F) sections. (A) *B. incompta* (sect. *Gomphostigma*) showing very numerous vessels almost always in contact with other vessels, many narrow rays, a single wider ray on the left, and weakly marked growth ring boundary in centre; (B) Growth ring boundary marked by 3–9 rows of radially flattened fibres and a shift from narrow latewood vessels to wide earlywood vessels in *B. nivea* (sect. *Alternifoliae*); (C) *B. albiflora* (sect. *Alternifoliae*) with growth ring marked in a similar fashion to *B. nivea*, but with a wider band of radially flattened fibres; (D) Few bi- to tetraseriate rays and very numerous short, uniseriate rays resembling axial parenchyma characteristic of *B. incompta* (sect. *Gomphostigma*); (E) *B. albiflora* (sect. *Alternifoliae*) with a similar proportion of uniseriate and multiseriate rays; (F) *B. forrestii* (sect. *Alternifoliae*) showing a few rays and multiple tracheids with fine helical thickenings. Scale bars: A = 500 µm, B–F = 100 µm.

Wood is diffuse porous in *B. fallowiana*, *B. myriantha* and *B. tubiflora*, and ring-porous to semi-ring-porous in all remaining species (Fig. 10A–C). Growth ring boundaries are absent (*B. fallowiana*, *B. tubiflora*; *B. myriantha* probably due to young age) or distinct, marked by tangential rows of radially flattened fibres (8–21 rows in *B. albiflora*, 1–3 in *B.* cf. *curviflora*, 3–7 in *B. colvilei*, 1–3 in *B. crispa*, 4–8 in *B. forrestii*, 2–5 in *B. globosa*, 1–3 in *B. incompta*, 1–10 in *B. nivea*, ≤3 in *B. paniculata*, 2–4 in *B.* x *wardii*, 1–3 in *B.* x *weyeriana*; Fig. 10A–C) or only by change in vessel diameter from narrow vessels in latewood to wider in earlywood (*B. crotonoides*).

Vessels are circular to oval in most species or mostly oval (in *B. incompta* and *B. myriantha*; Fig. 10A); slightly angular in outline in all species except *B. myriantha* where they are more rounded; narrow to very narrow [(16–)19–38(–50) µm wide] and numerous [(112–)125–375(–496) vessels mm^2^] in all species except for *B. incompta*, where they are very numerous (mean 955 vessels mm^-2^). Vessels are partly solitary (in most species < 40% of solitary vessels; in *B. incompta* all vessels are in contact with other vessels). They are disposed in small clusters and radial multiples usually of 2–4. There is no distinct pattern of vessel disposition, except for a weak tendency towards diagonal pattern in latewood of *B. albiflora* (Fig. 10C), *B. globosa* and *B. paniculata*.

Vessel elements are (55–)246–435(–1256) µm long. Perforation plates are exclusively simple. Intervessel pitting is alternate with pits mostly circular and rounded, sometimes polygonal (often in *B. albiflora*, rarely in other species), small to medium [(3.2–)4.2–6.9(–7.1) µm in vertical and (4.4–)4.6–8.2(–8.5) µm in horizontal diameters]. Apertures are slit-like (most species), lens-like (*B. fallowiana*, in *B. nivea* sometimes also circular) or short and almost circular (*B. crotonoides*, *B. fallowiana*, *B. globosa*, *B.* x *weyeriana*). Vessel–ray pits are similar in size and shape to intervessel pits, except for *B. incompta* where pitting is often scalariform or gash-like. Vessel-ray pits have clearly reduced borders (pitting in *B. paniculata* and *B.* x *weyeriana* could not be clearly observed). Very fine helical thickenings are present in most vessels and throughout their length in all species except *B.* x *wardii* (mostly in narrower vessels) and for *B. incompta* (completely missing). Numerous vascular tracheids are present in *B. albiflora*, *B. forrestii*, and *B. colvilei*, while fibriform vessels (*sensu* (Carlquist 2001)) were observed in *B.* x *wardii* and *B.* x *weyeriana*; in all these cases the cells have helical thickenings similar to those in vessels.

Fibres are libriform, mostly non-septate (septate fibres in *B.* cf. *curvifora* and *B. paniculata*), and thin- to thick-walled with simple to minutely bordered pits (Fig. 10D–F). Fibres are (45–)311–867(–2348) µm long. Axial parenchyma is absent or occasionally scanty paratracheal in solitary strands (*B. paniculata* and *B.* x *weyeriana*). In *B. incompta*, numerous parenchymatic cells observed in tangential longitudinal sections may either be solitary axial parenchyma cells or small, uniseriate rays (Fig. 10D).

Rays are uni-, bi- to tetraseriate (Fig. 10D–F). Mean number of uniseriate rays in most species is ≤4.2 mm^–1^ (in *B. incompta* ≤18.8 mm^–1^; Fig. 3D), and 2.2–8.4 mm^–1^ for multiseriate ones (usually ≤5.5 mm^–1^). Uniseriate rays are composed of square and upright cells in variable proportions. Multiseriate rays consist of rows of procumbent cells (≤12 rows in *B. albiflora*, ≤6 in *B. nivea*) sometimes mixed with square cells and flanked by 1–4 rows of square and upright cells; of square, upright, and procumbent cells intermixed throughout the ray body (*B. crispa*, *B. paniculata*, *B.* x *wardii*, *B.* x *weyeriana*); or mostly of square and upright cells intermixed with rare procumbent cells (*B.* cf. *curviflora*, *B. colvilei*, *B. crotonoides*, *B. fallowiana*, *B. forrestii*, *B. globosa*, *B. incompta*, *B. tubiflora*).

Upright and square, rarely also procumbent, ray cells of *B. globosa* and *B. tubiflora* contained prismatic crystals, and in ray cells of *B. fallowiana*, *B. forrestii*, *B. nivea*, *B. paniculata* and *B.* x *wardii* droplets of tannins were found.

## Discussion

### Relationships between micro- and macroscopic bark structure

In *Buddleja*, section *Gomphostigma* is like the sister group of the genus (*Freylinia*) in bark appearance and anatomy, and dissimilar to all remaining sections. Their species have a smooth, non-peeling, little or non-fissured bark surface, and homogenous texture. Instead, other *Buddleja* species share distinctly fissured and peeling bark with loose texture (Fig. 4). The barks of sect. *Gomphostigma* resemble the smooth or the shallow fissured type of Whitmore’s classification (1962a), whereas the latter ones belong to the scaly type.

These contrasting bark appearances correspond to the different modes of periderm formation. Multiple narrow bands of thin-walled phellem cells formed by deeply initiated phellogens act as separation layers between the bark scales occurring in most *Buddleja* species (Figs. 4, 8; Appendix B). This mode of sloughing is associated with collapsing and sclerification of secondary phloem performing the protective role instead of the periderm. Similar anatomies are found in other taxa having stringy peeling bark: *Lonicera* (Caprifoliaceae), *Melaleuca* (Myrtaceae), *Dodonaea* (Sapindaceae) (Chiang and Wang 1984; Eryomin and Kopanina 2012; Crivellaro and Schweingruber 2013; Schweingruber *et al.* 2013a, 2019). However, in *Vitis* (Vitaceae) (Eryomin and Kopanina 2012; Crivellaro and Schweingruber 2013; Schweingruber *et al.* 2013a, 2019) and in *Eucalyptus* (Myrtaceae) with stringy bark (Chattaway 1955), the division of labour is inversed: expanded, thin-walled cells of secondary phloem make separation layers, while thicker-walled phellem cells perform the protective function (Chattaway 1955).

Thus, stringy/scaly barks of similar appearance can arise from two different microstructural ground plans: either with (1) periderms (phellem) acting as separation layers combined with protective secondary phloem (*Lonicera*, most *Buddleja*; Fig. 8), or with (2) the secondary phloem (phloem parenchyma) performing the separation function, and the periderm responsible for the protective function (*Vitis*). Theoretically two other modes are possible: (3) both functions can be performed exclusively by secondary phloem, or (4) only by periderm. We did not find, however, any data supporting the existence of these options.

In contrast, the smoothness of non-peeling barks in sect. *Gomphostigma* and in the genus *Freylinia* is associated with a limited number and superficial origin of the periderms serving a protective function (Fig. 5D–F). This pattern of periderm initiation is found in many plants with smooth to shallow fissured barks, e.g. *Syringa* and *Fraxinus* (Oleaceae). Also, the lenticels found in *F. lanceolata* and *B. virgata* are common in species with non-scaly barks (Eryomin and Kopanina 2012; Crivellaro and Schweingruber 2013). Among four studied species with smooth barks, *F. tropica* is distinctive in showing shallow fissures with a tendency to form chunky scales (Fig. 4). This species also has discontinuous tangential bands of sclereids in its nonconducting secondary phloem. *Freylinia lanceolata* however has fewer sclereids arranged into small clusters, whereas *B. virgata* and *B. incompta* show no sclerification in their nonconductive secondary phloem (Frankiewicz *et al.* 2020). Therefore, the shallow fissured bark of *F. tropica* is likely associated with the abundance and arrangement of sclereids in its secondary phloem.

### *Taxonomic significance of bark anatomy in* Buddleja

Bark structure is different in the two constituents of sect. *Gomphostigma* than in other species. *Buddleja incompta* is very similar to *B. virgata* (Frankiewicz *et al.* 2020): the first periderm forms in the outer cortex, it produces uni- to biseriate phelloderm and thin-walled phellem, phloem does not undergo sclerification. The former species is distinctive only in the occurrence of rhytidome. In contrast, in all other *Buddleja* species, the first phellogen is deep-seated, it produces phellem and phelloderm (undergoing rapid obliteration), and cut-off phloem undergoes sclerification—a feature not reported outside *Buddleja* (Frankiewicz *et al.* 2020) (Fig. 4). Frankiewicz *et al.* (2020) incorrectly reported that the sclerification starts simultaneously or after initiation of periderm, but the order is reversed.

Stem and bark anatomy were used as taxonomic signals for *Buddleja* by Solereder (1908, pp. 538–547). Back then, the five genera presently classified in *Buddleja* (*Buddleja*, *Chilianthus*, *Emorya*, *Gomphostigma*, *Nicodemia*) were treated in the subfamily Buddleioideae within Loganiaceae. Solereder listed three diagnostic characters for Buddleioideae: (1) lack of intraxylary phloem, (2) presence of branched and glandular trichomes, and (3) pericyclic origin of the first periderm. The first feature differentiated Buddleioideae and Loganioideae – a group of species now known to be distantly related to *Buddleja* – and we can confirm that intraxylary phloem never occurs in *Buddleja*, although it is rarely found in other Scrophulariaceae (*Scrophularia*) (Doležal *et al.* 2018, pp. 379–380). Our results also corroborate the common presence of trichomes in *Buddleja*, and the few glabrous stems in our study may be incidental. It is also worth mentioning that Solereder distinguished *Gomphostigma* from other Buddleioideae using details of trichome cell wall structure: in the latter, the cell wall dividing trichomes in half is straight, in *Gomphostigma* it is undulating. We cannot agree with Solereder’s statement that in all Buddleioideae the first phellogen originates internally from pericyclic fibres. This initiation site is typical for most *Buddleja*, but not the sect. *Gomphostigma*.

Neither Solereder nor other early authors (Metcalfe and Chalk 1957) mentioned the abundant sclerification of phloem present in all *Buddleja* (save for sect. *Gomphostigma*). This feature was pointed out by Schweingruber *et al.* (2013b p. 263), who incorrectly identified sclerified cells as fibres, instead of sclerified cut-off phloem.

Scrophulariaceae are poorly studied anatomically: most species hitherto examined actually belong in Orobanchaceae or Plantaginaceae (Goldblatt and Manning 2002; Manning and Goldblatt 2012, pp. 731–763). Precise descriptions are absent, and detailed photos of bark anatomy are available only for *Freylinia* (Frankiewicz *et al.* 2020), *Scrophularia* (Makbul *et al.* 2006; Schweingruber and Landolt 2010; Schweingruber *et al.* 2013b pp. 264–266; Doležal *et al.* 2018, pp. 379–380), and *Verbascum* (Schweingruber and Landolt 2010; Schweingruber *et al.* 2013b, pp. 267–270). In these taxa, sieve tubes are often in small groups. The transition from conducting to nonconducting phloem may be marked by diffuse sclerification varying in intensity, from few (e.g. *S. latifolia*) to numerous, thick-walled sclerified cells (e.g. *V. arcturus*). Nevertheless, none of the examined species undergoes nearly solid phloem sclerification as reported in most *Buddleja* species, and the most similar condition is found in *F. tropica* (but not in *F. lanceolata*). Conversely, three of nine *Scrophularia* species and seven of nine *Verbascum* species in the Xylem Database (Büntgen *et al.* 2014) are completely devoid of phloem sclerification. Available sources do not allow for identification of the initiation site of the first phellogen in most taxa: it is the outer cortex in *Freylinia*, and probably likewise in at least some *Scrophularia* (*S. parviflora*) and *Verbascum* (*V. arcturus*). In all other studied members of Scrophulariaceae, phellem consists of thin-walled (phelloid) cells, and phloem and ray dilatation occurs commonly.

Based on this, admittedly limited, evidence, we hypothesise that moderate phloem sclerification and subepidermal periderm initiation may be plesiomorphic for Scrophulariaceae. Such anatomy makes a convenient starting point for evolution of both phloem completely devoid of sclerification in sect. *Gomphostigma* and phloem undergoing nearly solid sclerification in the remaining clades of *Buddleja*, supporting the position of sect. *Gomphostigma* as sister to the rest of the genus (Fig. 1). The alternative scenario resolved in certain molecular analyses (Chau *et al.* 2017, 2018), with sect. *Gomphostigma* and sect. *Salviifoliae* swapped, requires evolution of abundant sclerification of phloem and deep-seated periderm initiation in the most recent common ancestor of *Buddleja* and then a complete loss of sclerification combined with a change in the phellogen initiation site in sect. *Gomphostigma*—a less parsimonious scenario.

Our examination of semi-thin sections of *B. saligna* showed that uni- and biseriate phelloderm is present in this species (Fig. 6C, D). It is prominent in young periderms and becomes obliterated during bark transformations. Frankiewicz *et al.* (2020) did not recognize phelloderm in the periderm of *Buddleja* species (except sect. *Gomphostigma*) due to its obliteration.

### *Taxonomic significance of wood anatomy in* Buddleja

The newly examined species fall within the range of anatomical diversity reported for *Buddleja* (Frankiewicz *et al.* 2020). Three earlier attempts to identify wood characters useful for delineation of infrageneric sections were unsuccessful: qualitative trait variation is limited in *Buddleja* (Carlquist 1997), and quantitative traits correlate with plant size rather than phylogenetic relationships (Terrazas *et al.* 2008; Frankiewicz *et al.* 2020).

Scarcity or absence of axial parenchyma is universal among *Buddleja*, helical thickenings were absent only in *B. utahensis* (Carlquist 1997), *B. virgata* (Frankiewicz *et al.* 2020) and *B. incompta* (this study). The two latter species—the sole members of sect. *Gomphostigma*—are also characterised by a markedly greater number of uniseriate rays mm^-1^ than in any other *Buddleja* (mean: 14.8 and 18.8, respectively; in most other species it typically is ≤5.6 mm^–1^). These rays are often short and resemble short strands or fusiform solitary cells of axial parenchyma. Both species of sect. *Gomphostigma* are also distinct in a very high number of vessels mm^–2^ (≤679 mm^––2^ in *B. virgata*, 955 mm^–2^ in *B. incompta*; in other species it rarely exceeds 400 mm^–2^), which in turn leads to the low percentage of solitary vessels. Consequently, the combination of high vessel density, vessels contacting other vessels, very numerous short and uniseriate rays resembling axial parenchyma, and lack of helical thickenings can distinguish sect. *Gomphostigma* from the other sections of *Buddleja*.

### *Consequences for taxonomy of* Buddleja

The anatomical traits of southern African *Buddleja* sect. *Gomphostigma* may represent the plesiomorphic state for the genus, since they differ from those observed in the rest of the genus but share many similarities with traits in *Freylinia* species in tribe Teedieae (the sister to *Buddleja*). This supports sect. *Gomphostigma* as sister to the rest of *Buddleja*. Phylogenetic analyses of high-throughput sequencing of loci from targeted sequence capture resolved sect. *Gomphostigma* as sister to the rest of the genus with very strong support (Chau *et al.* 2018), although analyses with different smaller molecular datasets placed it in other positions with weak support (Chau *et al.* 2017, 2018). The rather unique reproductive morphology of sect. *Gomphostigma*, including racemose inflorescences, cup-shaped corollas and exserted stamens (Chau *et al.* 2017), may represent autapomorphies for the section or ancestral trait states for the genus, thus being of little taxonomic use.

## Conclusions

The relationship between the bark micro- and macrostructure is far from straightforward. Smooth bark surface is related to a small number of periderms, their superficial origin, and limited sclerification. This combination allows for the development and retention of conspicuous lenticels. Instead, fissured, stringy/scaly barks require division of labour: some tissues take a protective function, while others serve as separation layers. In the case of *Buddleja*, the former is done by sclerified phloem, while the thin-walled phellem serves the latter function. The literature shows that the reverse division of labour is also possible. Theoretically, both functions could be served by the same tissue, but no evidence of such cases could be found.

Bark—and to a lesser degree wood—anatomy support a sister relationship between South African *Buddleja* sect. *Gomphostigma* and all remaining *Buddleja* species. This shows that anatomy can be a useful source of data to complement molecular phylogenies, especially when molecular markers alone do not provide satisfactory resolution (total evidence approach).

## Supporting Information

The following additional information is available in the online version of this article

**Appendix A** – Accession information for specimens of *Buddleja* used in the study

**Appendix B** – Tabulated summary of bark anatomical and morphological traits in *Buddleja* and *Freylinia*.

plad003_suppl_Supplementary_Appendix_A

plad003_suppl_Supplementary_Appendix_B

## Data

Photos of herbarium vouchers, close-ups of bark surface, and measurements of quantitative anatomical traits are available on FigShare DOI: 10.6084/m9.figshare.14798034
