# Supplementary material for: Wood and bark structure in Buddleja: anatomical background of stem morphology
Source: AoB Plants. 2023 Jan 24;15(2):plad003. doi: 10.1093/aobpla/plad003 (PMC10060081; doi:10.1093/aobpla/plad003)
Supplement: plad003_suppl_Supplementary_Appendix_A [file plad003_suppl_supplementary_appendix_a.pdf]

Appendix A

Appendix A. Sample information for specimens used in the study grouped taxonomically following conclusions of Frankiewicz *et al.* (2020) and Chau *et al.* (2017). Stem diameter (∅) of samples are provided under Specimen Description. For newly examined species, voucher information and additional specimen descriptions are provided. Specimens re-examined after Frankiewicz *et al.* (2020) are indicated. All newly examined species except for *B. incompta* (KF027) and *B. saligna* (AO489-21) were cultivated in The National Buddleja Collection at Longstock Park Nursery (Stockbridge, Hampshire, United Kingdom) and sampled on 25.07.2020. Herbarium acronyms follow Thiers (2013).

Taxonomy	Species	Specimen ID: Voucher	Specimen description	Remarks
Outgroup	*Freylinia lanceolata* (L.) G.Don	KF007	∅ 18.2 mm	Re-examined
	Freylinia tropica S.Moore	KF008	∅ 21.8 mm	Re-examined
Sect. *Gomphostigma*	*Buddleja incompta* L.f.	KF027: NBG1465285 (John C. Manning 3447: South Africa, Northern Cape, Sutherland, Bo-Visrivier road near Blesfontein Farm; July 2013)	Cane shrub, sampled lateral branch ∅ 12.1 mm	Originally labelled as *Gomphostigma incomptum*. Also embedded in GMA.
	Buddleja virgata L.f.	KF010	∅ 10.0 mm	Re-examined
	Buddleja virgata L.f.	KF011	∅ 21.2 mm	Re-examined
Sect. *Salviifoliae*	*Buddleja salviifolia* (L.) Lam.	KF001	∅ 15.5 mm	Re-examined
	Buddleja salviifolia (L.) Lam.	KF013	∅ 14.5 mm	Re-examined
Sect. *Chilianthus*	*Buddleja auriculata* Benth.	KF003	∅ 14.0 mm	Re-examined
	Buddleja auriculata Benth.	KF012	∅ 16.2 mm	Re-examined

Taxonomy	Species	Specimen ID: Voucher	Specimen description	Remarks
	Buddleja dysophylla (Benth.) Radlk.	KF009	∅ 25.9 mm	Re-examined
	Buddleja glomerata H. Wendl.	KF006	∅ 23.9 mm	Re-examined
	Buddleja saligna Willd.	AO489-21: JRAU (Alexei A. Oskolski: South Africa, Gauteng, Johannesburg, Auckland Park Kingsway campus of the University of Johannesburg, along Kingsway; 11 Aug 2021)	Prostrated shrub ca. 2.5 m tall, pale flowers in terminal panicles; sampled lateral branch; cultivated ∅ 10.0 mm	Wood and bark anatomy of this species was previously described by Frankiewicz *et al.* (2020) and new specimen was consulted for the purpose of this paper. Embedded in GMA.
	Buddleja saligna Willd.	KF002	∅ 23.92 mm	Re-examined
Sect. *Alternifoliae*	*Buddleja albiflora* Hemsl.	KF033: WA0000085497	Shrub ca. 2–3 m tall, flowering at time of sampling; sampled lateral branch ∅ 21.3 mm	
	Buddleja cf. *curviflora* Hook. & Arn.	KF044: WA0000085504	Sampled lateral branch ∅ 10.6 mm	Originally labelled as *B. myriantha*.
	Buddleja colvilei Hook.f.	KF041: WA0000085510	Sampled lateral branch ∅ 7.5 mm	
	Buddleja crispa Benth.	KF042: WA0000085508	Shrub ca. 1.5 m tall, no flowers at time of sampling, previously grown in a pot, transplanted to	Originally labelled as *B. farreri*.

Appendix A

Taxonomy	Species	Specimen ID: Voucher	Specimen description	Remarks
			ground, but may not survive winter; lateral branch ∅ 7.4 mm	
	Buddleja crispa Benth.	KF046: WA0000085502	Shrub ca. 0.5 m tall; sampled lateral branch ∅ 8.5 mm	Originally labelled as *B. tibetica*.
	Buddleja fallowiana Balf.f. & W.W.Sm.	KF037: WA0000085509	Shrub ca. 0.5–1 tall, branches covered with thick, woolly trichomes, no flowers at time of sampling; sampled lateral branch ∅ 3.1 mm	Not studied morphologically due to limited periderm development.
	Buddleja forrestii Diels	KF017	∅ 7.6 mm	Re-examined
	Buddleja forrestii Diels	KF034: WA0000085501	Shrub ca. 1.5 m tall, flowering at time of sampling; sampled lateral branch ∅ 12.5 mm	Originally labelled as *B. limitanea*. This species was previously included in Frankiewicz *et al.* (2020).
	Buddleja forrestii Diels	KF040: WA0000085512	Sampled lateral branch ∅ 8.2 mm	Originally labelled as *B. pterocaulis*. This species was previously included in Frankiewicz et al. (2020).
	Buddleja lindleyana Fortune	KF015	∅ 16.3 mm	Re-examined
	Buddleja myriantha Diels	KF030: WA0000085500	Shrub ca. 0.5 m tall; sampled lateral branch ∅ 3.0 mm	Only transverse sections were available.

Taxonomy	Species	Specimen ID: Voucher	Specimen description	Remarks
				Not studied morphologically due to limited periderm development.
	Buddleja nivea Duthie	KF035: WA0000085505	Shrub ca. 2 m tall, branches covered with thick, woolly trichomes, flowering at time of sampling; sampled lateral branch ∅ 11.9 mm	
	Buddleja paniculata Wall.	KF036: WA0000085506	Shrub ca. 4–5 m tall, branches covered with thick, woolly trichomes, no flowers at time of sampling, but flowers regularly; sampled lateral branch ∅ 16.3 mm	
Sect. *Buddleja*	*Buddleja aromatica* J.Rémy	KF014	∅ 5.6 mm	Re-examined
	Buddleja cordata Kunth	KF005	∅ 40.0 mm	Re-examined
	Buddleja coriacea J.Rémy	KF016	∅ 15.8 mm	Re-examined
	Buddleja crotonoides A.Gray	KF043: WA0000085507	Shrub ca. 1.5 m tall, dried flowers present at time of sampling, previously grown in a pot, transplanted to ground, but may not survive winter; sampled lateral branch ∅ 5.6 mm	Originally labelled as *B. crotonoides amplexi caulis*.

Appendix A

Taxonomy	Species	Specimen ID: Voucher	Specimen description	Remarks
	Buddleja globosa Hope	KF031: WA0000085499	Shrub ca. 0.75 m tall; sampled lateral branch ∅ 4.0 mm	Only transverse sections were available, they were studied for bark anatomy.
	Buddleja globosa Hope	KF032: WA0000085498	Shrub ca. 1 m tall, no flowers at time of sampling, 'lemon ball' variety; sampled lateral branch ∅ 18.2 mm	
	Buddleja longiflora Brade	KF018	∅ 13.9 mm	Re-examined
	Buddleja tubiflora Benth.	KF045: WA0000085503	Shrub ca. 1.5–2 m tall, flowering at time of sampling; sampled lateral branch ∅ 5.0 mm	
Hybrids	*Buddleja* x *wardii* C.Marquand	KF039: WA0000085513	Shrub ca. 2 m tall, no flowers at time of sampling; sampled lateral branch ∅ 8.3 mm	A hybrid of: *B. alternifolia* (*Alternifoliae*) and *B. crispa* (*Alternifoliae*)
	Buddleja x *weyeriana* Weyer ex Bean	KF038: WA0000085511	Sampled lateral branch ∅ 10.8 mm	A hybrid of: *B. davidii* (*Alternifoliae*) and *B. globosa* (*Buddleja*)

Literature

Chau JH, O'Leary N, Sun WB, Olmstead RG. **2017**. Phylogenetic relationships in tribe Buddlejeae (Scrophulariaceae) based on multiple nuclear and plastid markers. *Botanical Journal of the Linnean Society* **6**: 137–166.

Frankiewicz KE, Chau JH, Oskolski AA. **2020**. Wood and bark of *Buddleja*: uniseriate phellem, and systematic and ecological patterns. *IAWA Journal* **42**: 3–30.

Thiers BM. **2013**. *(continuously updated) Index Herbariorum: a global directory of public herbaria and associated staff. New York Botanical Garden's Virtual Herbarium*.
